# A Computationally Inexpensive Optimal Guidance via Radial-Basis-Function Neural Network for Autonomous Soft Landing on Asteroids

**DOI:** 10.1371/journal.pone.0137792

**Published:** 2015-09-14

**Authors:** Peng Zhang, Keping Liu, Bo Zhao, Yuanchun Li

**Affiliations:** 1 Department of Control Science and Engineering, Jilin University, Changchun, Jilin, China; 2 Department of Control Engineering, Changchun University of Technology, Changchun, Jilin, China; 3 State Key Laboratory of Management and Control for Complex Systems, Institute of Automation, Chinese Academy of Sciences, Beijing, China; Jiangnan University, CHINA

## Abstract

Optimal guidance is essential for the soft landing task. However, due to its high computational complexities, it is hardly applied to the autonomous guidance. In this paper, a computationally inexpensive optimal guidance algorithm based on the radial basis function neural network (RBFNN) is proposed. The optimization problem of the trajectory for soft landing on asteroids is formulated and transformed into a two-point boundary value problem (TPBVP). Combining the database of initial states with the relative initial co-states, an RBFNN is trained offline. The optimal trajectory of the soft landing is determined rapidly by applying the trained network in the online guidance. The Monte Carlo simulations of soft landing on the Eros433 are performed to demonstrate the effectiveness of the proposed guidance algorithm.

## I. Introduction

Over the past few years, there was a strong interest in sending robotic spacecraft to asteroids in our solar system. Due to its high scientific value, more and more missions focus on the soft landing of the spacecraft on the asteroid’s surface. As asteroids are far from the earth, communications between the spacecraft near asteroids and the control center on the earth will have a long delay. Hence, landing on asteroids must be done autonomously using on-board algorithms. The autonomous Guidance, Navigation, Control (GNC) technology is essential and becomes a big challenge to ensure the safety of soft landing mission.

Many studies on the autonomous guidance and control of the soft landing on asteroids have been carried out. Guelman et al. [1] researched the final approach phase and vertical landing on a spherical asteroid with a power limited, electrically propelled spacecraft. Based on the autonomous navigation system using the feature tracking technology, Huang et al. [2] planned desired descent landing trajectories of the spacecraft as the three power polynomial form with the initial and terminal constraint, and then applied a variable structure control to track the trajectory. This reference trajectory guidance based on the three power polynomial technology was widely applied [3–5]. Roberto Furfaro et al. [6] developed a novel closed-loop autonomous guidance law based on multiple sliding surfaces for the soft landing of the spacecraft on the designated point on the asteroid. In these previous contributions, the proposed autonomous guidance algorithms did not consider the optimality of the landing trajectory of the spacecraft.

In order to reduce fuel consumption and increase payload capacity, optimal guidance is essential for the soft landing on the asteroid. However, the analytical solutions of the optimal guidance are hardly figured out. Instead, numerical solutions are usually applied. Lantoine et al. [7] investigated a technique for computing optimal trajectories for soft landing in an irregular gravity field of a rotating asteroid. Zhang et al. [8] presented an optimization method based on the pseudo-spectral technology and the sequential quadratic programming for the minimum-energy soft landing on the asteroid. Wang et al. [9] investigated the fuel-optimal soft landing based on the Gauss pseudo-spectral method. However, the presented optimal guidance laws above are all with high computational cost due to the complicated model of the asteroid’s gravitational field, which may affect the real time performance of the autonomous soft landing. As the spacecraft hovers vary over a region of state space at the beginning of the soft landing, a fixed optimal control which was planned with a certain initial hovering point may fail. Due to the time delay, solving the optimization problem on the earth and transferring the result to the spacecraft is also impossible. Hence, a computationally inexpensive and real time autonomous optimal guidance is necessary. The traditional optimal guidance law needs to be improved to meet the requirements of the autonomous soft landing.

The indirect method is usually applied to solve the optimal guidance problems. Based on the optimal control determined by the Pontryagin Principle, the optimization problem is transformed into a TPBVP and solved by the numerical methods such as Newton’s or Powell’s [10–12]. Then, the optimal soft landing trajectory can be determined. The main advantage of the indirect method lies in that the optimal trajectory and control can be determined by several initial co-states. Most of computational and time cost in the indirect method are consumed in solving the shooting equations. Hence, if the desired initial co-states can be quickly and easily obtained, the optimal guidance law based on the indirect method can be applied in the autonomous soft landing.

The improvement of the functional approximation technology of the neural network gives an opportunity to implement the realtime autonomous optimal guidance [13–16]. Cheng et al. [17] proposed a fixed-final time optimal control law using neural networks and Hamilton-Jacobi-Bellman (HJB) equations for general affine in the input nonlinear systems. Medagam et al. [18] presented a nonlinear optimal control technique based on approximating the solution to the HJB equation by the RBFNN Hossain et al. [19] presented an investigation into the challenges in implementing a hard real-time optimal non- stationary system using the general regression neural network. Because of its excellent approximation properties [20, 21], the RBFNN was widely applied since be introduced in 1988 [22]. It is applied to improve the indirect optimization method in our work.

An optimal guidance algorithm for autonomous soft landing on asteroids was proposed in this paper. The optimization problem of the soft landing trajectory is formulated, and then transformed into a TPBVP through the optimal control obtained by the Pontryagin Principle. The RBFNN, which was trained by the database on the earth, was applied online to determine the optimal trajectory of the soft landing. A normalization method is applied to ensure the approximation property of the RBFNN. Monte Carlo simulations are performed to analyze the performance of the proposed optimal guidance. The main contributions of this work are presented as follows: (i) it’s feasible to develop an autonomous optimal guidance algorithm via RBFNN for the spacecraft soft landing on the asteroid. (ii) the application of the RBFNN in the indirect optimal method is investigated.

The rest of the paper is organized in the following form. In Sec II, the optimization problem of the soft landing trajectory is formulated. The orbital dynamics of the spacecraft and the gravitational field of the asteroid are modeled. In Sec III, the optimization problem is transformed into a TPBVP through the optimal control determined by the Pontryagin Principle. Then, the optimal guidance via the RBFNN is proposed. In Sec IV, Monte Carlo simulations are performed to verify the effectiveness of the proposed algorithm. Ultimately, the conclusion follows in Sec V.

## II. Problem formulation

**Fig 1:**
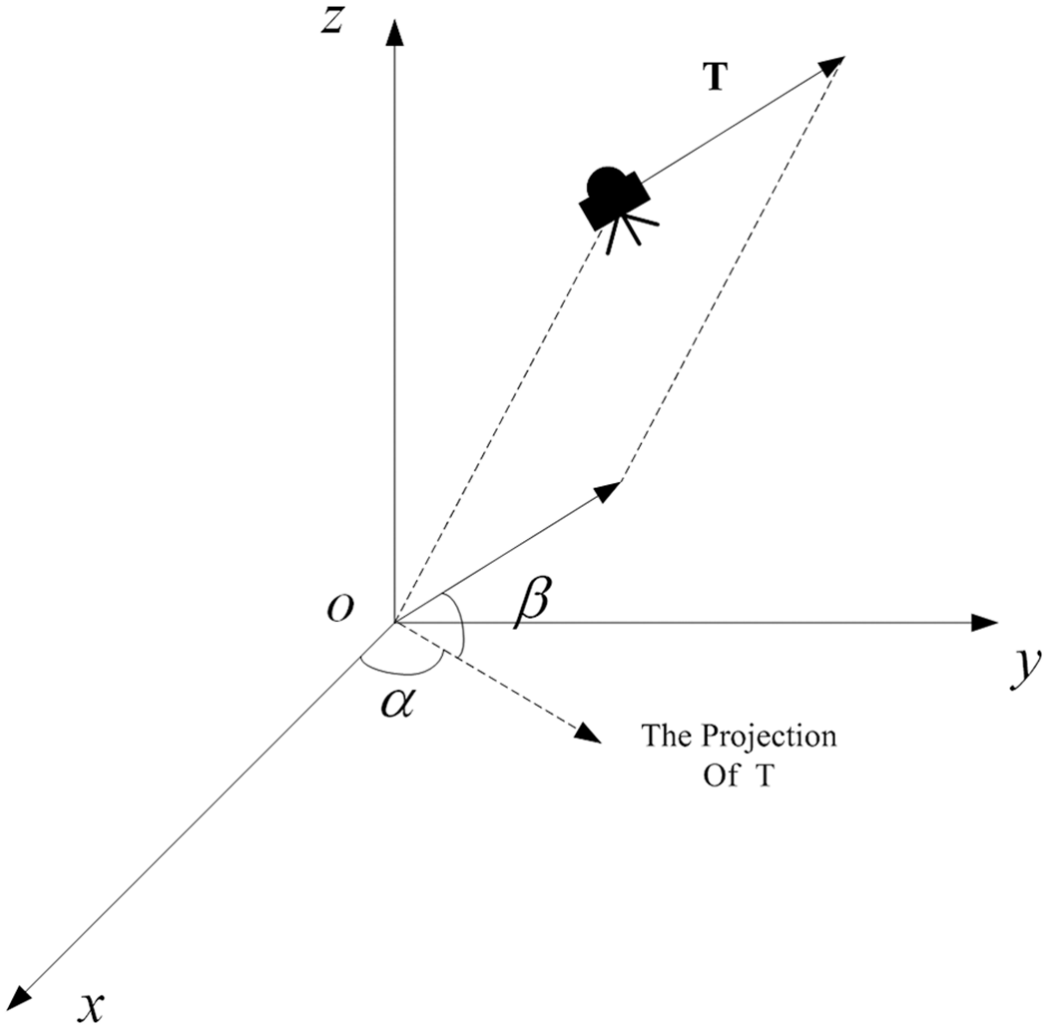
The conceptual illustration of the spacecraft’s thrust vector. This figure illustrates the physical relationships of the variables in Eq (1).

The following assumptions need to be declared before the problem formulation. The rotation rate of the asteroid is time invariant. The spacecraft’s soft landing starts from a hovering point, and ends with a specified location on the asteroid’s surface. The initial position, velocity, and mass of the spacecraft can be obtained by the navigation system. The final position and velocity are determined to meet mission requests. As shown in Fig 1, the spacecraft is controlled by a main thruster. The magnitude and direction of the spacecraft’s thrust are adjustable. The solar radiation pressure is not considered, it has a significant contribution only for very small asteroids with low gravity field [7].

### The trajectory optimization problem

The orbital dynamic equations of the spacecraft near the asteroid were widely researched [23]. Aiming to formulate the trajectory optimization problem of the soft landing, dynamic equations of the spacecraft are established in the body-fixed frame [3, 4]. The origin of the body-fixed frame coincides with the mass center of the asteroid. The z-axis coincides with the spin axis of the asteroid, and the x-axis coincides with minimum inertia axis of the asteroid. The y-axis can be defined by the right-hand rule.

In the body-fixed frame, the orbital motion of the spacecraft can be expressed as

$$\begin{array}{c} \left\{ \begin{array}{c} \ddot{x}=\omega^{2}x+2\omega \dot{y}+g_{x}+T\cos\beta \cos\alpha /m\\ \ddot{y}=\omega^{2}y-2\omega \dot{x}+g_{y}+T\cos\beta \sin\alpha /m\\ \ddot{z}=g_{z}+T\sin\beta /m\\ \dot{m}=-T/I_{s}g_{0} \end{array}\;\right. \end{array}$$ (1)

where

*x*, *y*, *z* are the position of the spacecraft.

*g*_*x*_, *g*_*y*_, *g*_*z*_ are the gravitational acceleration of the asteroid in three axis.

*m* is the mass of the spacecraft.

*ω* is the angular velocity of the asteroid’s rotation.

*T* is the magnitude of the spacecraft’s thrust vector, which is limited to the domain from zero to *T*_max_.

*β* is the angle between the thrust vector and its projection on the o-x-y plane,

*α* is the angle between the projection and the direction of the x axis.

*I*_*s*_ is the specific impulse of the thruster,

*g*_0_ is the gravitational acceleration on the earth.

Define the state vector as $\text{x}={\left[x,y,z,\dot{x},\dot{y},\dot{z},m\right]}^{T}$, and the control vector as **u** = [*T*, *α*, *β*]^*T*^. Then Eq (1) can be converted into a nonlinear differential equation as

$$\begin{array}{c} \dot{x}=f\left(x,u\right) \end{array}$$ (2)

The cost function is expressed as

$$\begin{array}{c} J\left(\cdot \right)=\int_{t_{0}}^{t_{f}}T^{2}dt \end{array}$$ (3)

Hence, the trajectory optimization problem of the soft landing can be expressed as

$$\begin{array}{c} \begin{array}{c} \min\;J(\cdot )\\ s.t.\;\dot{x}=f\left(x,u\right)\\ \;T\in [0,T_{\max}] \end{array} \end{array}$$ (4)

with the hard boundary conditions as

$$\begin{array}{c} x\left(t_{0}\right)=[x_{0},\;y_{0},\;z_{0},\;{\dot{x}}_{0},\;{\dot{y}}_{0},\;{\dot{z}}_{0},\;m_{0}] \end{array}$$ (5)

$$\begin{array}{ccc} x(t_{f}) & = & x_{f},\;y\left(t_{f}\right)=y_{f},\;z\left(t_{f}\right)=z_{f},\\ \dot{x}\left(t_{f}\right) & = & 0,\;\dot{y}\left(t_{f}\right)=0,\;\dot{z}\left(t_{f}\right)=0, \end{array}$$ (6)

### The gravitational potential function of the asteroid

The gravitational acceleration **g** = [*g*_*x*_, *g*_*y*_, *g*_*z*_]^*T*^ in Eq (1) can be determined by the gravitational potential function of the asteroid, as

$$\begin{array}{c} g=\partial U/\partial r \end{array}$$ (7)

where

*U* is the gravitational potential function of the asteroid.

**r** = [*x*, *y*, *z*]^*T*^ is the position vector of spacecraft.

Due to the highly irregular shape, several models were developed to approach the non-spherical gravitational field of the asteroid. Among them, the spherical harmonic expansion model (SHEM) [24] and the constant-density polyhedron model [25], are widely applied in the autonomous GNC system for the soft landing. In the following part, they are introduced with a simple evaluation of the advantages and drawbacks. It should be mentioned that due to the limitation of the personal computer’s computational ability, the second-order expansion of the SHEM is selected for the simulation in Sec IV. Theoretically, the polyhedron model can also be applied in our optimal guidance algorithm.

#### The spherical harmonic expansion model (SHEM)

The SHEM divides the exterior gravitational potential of the asteroid into two parts, the spherical part and the irregular part. The irregular part is approached by an infinite series expansion in solid spherical harmonics. By the SHEM, the exterior gravitational potential of the asteroid can be expressed as

$$\begin{array}{c} U=\frac{GM}{r}\left(1+\sum_{i=1}^{N}{\sum_{j=0}^{i}(\frac{R_{0}}{r})}^{i}P_{i}^{j}\left(\sin\delta \right)\left[C_{ij}\cos\left(j\gamma \right)+S_{ij}\sin\left(j\gamma \right)\right]\right) \end{array}$$ (8)

where

*G* is the gravitational constant,

*M* is the total mass of the body,

*R*_0_ is the normalizing radius of the asteroid,

$P_{i}^{j}$ is the associated Legendre function,

*C*_*ij*_and *S*_*ij*_ are the spherical harmonic gravity coefficient,

*r*, *δ* and *γ* are the radius, latitude, and longitude of the field point.

The finite truncation of the SHEM is usually sufficient to get a good accuracy and easy to be used [4, 26]. However, the series of the SHEM may be divergent in the Brillouin sphere [24] of the asteroid.

#### The polyhedron model

A constant density polyhedron is applied to approach the irregular shape of the asteroid. In this model, the exterior gravitational potential of the asteroid can be expressed as

$$\begin{array}{c} U=\frac{1}{2}G\sigma \sum_{e\in edges}r_{e}\cdot E_{e}\cdot r_{e}\cdot L_{e}-\frac{1}{2}G\sigma \sum_{f\in faces}r_{f}\cdot F_{f}\cdot r_{f}\cdot \omega_{f} \end{array}$$ (9)

where

*σ* is the constant-density of the body,

**r**_*e*_ is a vector from the field point to an arbitrary point on each edge,

**r**_*f*_ is a vector from the field point to an arbitrary point on each face,

**E**_*e*_ is a dyad defined in terms of the face and edge normal vectors associated with each edge,

**F**_*f*_ is the outer product of face normal vectors,

*L*_*e*_ is a logarithmic term expressing the potential of a 1D straight wire,

*ω*_*f*_ is the solid angle subtended by a face when viewed from the field point.

The polyhedron model can formulate the exterior gravitational potential of the asteroid anywhere in space, not limited to the outside of the Brillouin sphere. However, it needs to sum over all the edges and all the faces of the polyhedron to compute the gravitation in one field point. It is a large computational burden, especially when the polyhedron is highly accurate.

## III. The realtime optimal guidance via RBFNN

### The relative TPBVP

First of all, the trajectory optimization problem of the soft landing is transformed to a TPBVP through the optimal control obtained by the Pontryagin Principle theory.

Combine Eqs (2) and (3), define a Hamiltonian function as

$$\begin{array}{c} H\left(x,u,t\right)=\lambda {\left(t\right)}^{T}f\left(x,u\right)+T^{2} \end{array}$$ (10)

where *λ*(*t*) = [*λ*_1_, *λ*_2_⋯, *λ*_7_] is the co-state vector.

According to the Pontryagin Principle theory, the optimal control is the one which can minimize the Hamiltonian function, as

$$\begin{array}{c} u^{*}=\underset{u\in \Omega }{\text{arg}\min}H\left(x^{*},u,t\right) \end{array}$$ (11)

where Ω is the feasible region of the control.

As the latitude and longitude angle of the thruster are unlimited, the optimal control α* and β* can be determined by the partial derivative of the Hamiltonian function, as

$$\begin{array}{c} \frac{\partial H}{\partial \alpha }=0,\;\frac{\partial^{2}H}{\partial \alpha^{2}}>0,\;\frac{\partial H}{\partial \beta }=0 \end{array}$$ (12)

Hence, the optimal control α* and β* can be expressed as Eqs (13) and (14).

$$\begin{array}{c} \alpha^{*}=\arctan(\frac{\lambda_{5}}{\lambda_{6}}),sign\left(\alpha^{*}\right)=-sign\left(\lambda_{4}\right) \end{array}$$ (13)

$$\begin{array}{c} \beta^{*}=\arctan(\frac{\lambda_{6}}{\lambda_{4}\cos\alpha^{*}+\lambda_{5}\sin\alpha^{*}}) \end{array}$$ (14)

As the magnitude of the thrust is limited to [0, *T*_max_], the optimal control T* is a piecewise function according to the Pontryagin Principle theory. Putting Eqs (13) and (14) into Eq (11), the optimal T* can be expressed as

$$\begin{array}{c} T^{*}=\{\begin{array}{c} T_{\max}\;\text{,}\;S\in \left(-\infty ,-1\right]\\ -\frac{1}{2}T_{\max}\left(S-1\right)\;,\;S\in \left(-1,1\right)\\ 0\;\text{,}\;S\in [1,+\infty ) \end{array} \end{array}$$ (15)

where *S* is the switching function, as

$$\begin{array}{c} S=1-\frac{\sqrt{\lambda_{4}^{2}+\lambda_{5}^{2}+\lambda_{6}^{2}}}{m}-\frac{\lambda_{7}}{I_{s}g_{0}} \end{array}$$ (16)

As shown in Eqs (13)–(16), the optimal control u* can be determined by the co-states *λ*(*t*). According to the Pontryagin Principle theory, the co-states meet the regular expression as

$$\begin{array}{c} \dot{\lambda }\left(t\right)=-\partial H\left(x,u,\;t\right)/\partial x\; \end{array}$$ (17)

As shown in Eq (6), the terminal position and velocity of the spacecraft are constrained. Hence, their relative final co-states are uncertain. However, the terminal mass of the spacecraft is unconstrained and the mass-relative final co-state can be determined as

$$\begin{array}{c} \lambda_{7}\left(t_{f}\right)=0 \end{array}$$ (18)

In summary, the optimization problem is transformed to an TPBVP, the Canonical equation of which is shown as

$$\begin{array}{c} \left\{ \begin{array}{c} \dot{x}=\partial H/\partial \lambda \\ \dot{\lambda }=-\partial H/\partial x \end{array}\right. \end{array}$$ (19)

Its boundary conditions are shown as Eqs (5), (6) and (18).

### The optimal guidance

To every initial co-states *λ*(*t*_0_), the relative final states **x**(*t*_f_) and co-states *λ*(*t*_f_) can be numerically integrated through Eq (19). Hence, the proposed TPBVP can be transformed into a set of nonlinear shooting equations as

$$\begin{array}{c} F\left(\lambda \left(t_{0}\right)\right)=\left[x^{\prime }\left(t_{f},\lambda \left(t_{0}\right)\right)-{x^{\prime }}_{f},\lambda_{7}\left(t_{f},\lambda \left(t_{0}\right)\right)\right]=0\; \end{array}$$ (20)

where ${\text{x}}^{\prime }={\left[x,y,z,\dot{x},\dot{y},\dot{z}\right]}^{T}$

Several methods can solve the shooting equations, such as the Newton’s and the Powell’s. However, all these methods need thousands of iterations, which result in their high computational complexities. Considering the optimal guidance based on the indirect method, most computational cost is consumed in the process of solving the shooting equations. Hence, the key problem is how to obtain the desired initial co-states in a computationally inexpensive way.

The solution of the equations can be treated as the map of the initial states **x**(*t*_0_) to the desired initial co-states *λ*(*t*_0_). It is difficult to deduce the analytical form of the map. However, the map may be approached by the functional approximation technology. In our research, the RBFNN is applied to make the indirect method available online. The spacecraft can acquire the initial co-states through the trained RBFNN quickly. Integrate Eq (19) forward with the initial states and co-states, the optimal control can be determined at each point along the trajectory.

**Fig 2:**
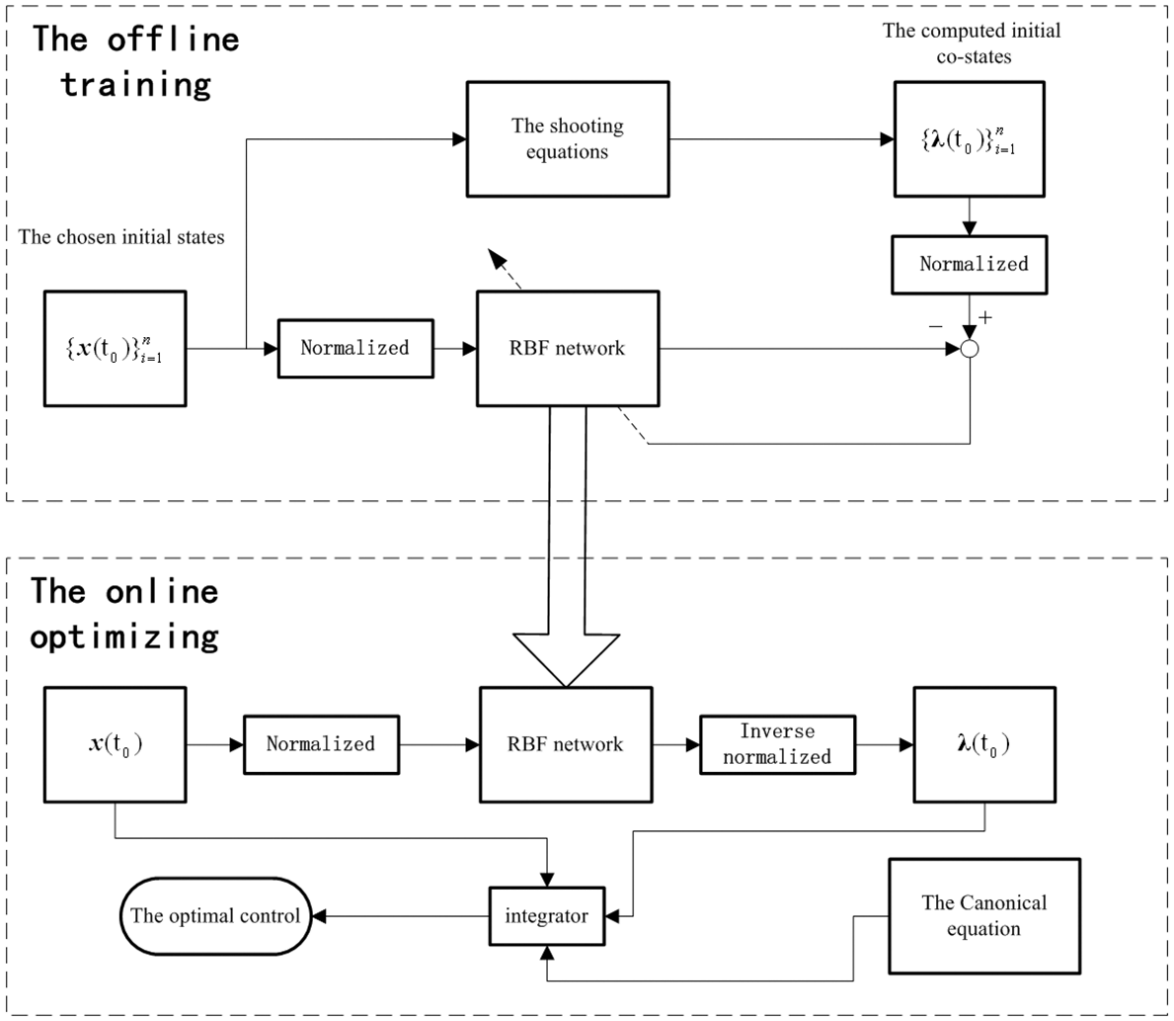
The structure of the optimal guidance. This figure illustrates the structure of the optimal guidance. The RBFNN is trained by the database obtained by solving the shooting equations. Then it is applied online to obtain the relative initial co-states quickly. Through the initial co-states, the optimal control can be determined by the analytical equations as Eqs (13)–(15).

The structure of the online optimal guidance is shown in Fig 2

Thousands sets of initial states and their relative initial co-states, which are acquired through solving the shooting equations by Newton’s method, establish the training database. Then, the RBFNN is trained by the database. The pre-computation to obtain the database is also very complex. However, it is an offline computation, which is on the earth and will not threaten the real time performance of the optimal guidance. The spacecraft can easily acquire the relative optimal control through the well trained network online.

#### The normalization method

The magnitude of each element in the states and co-states are quite different, which may threaten the approximation property of the RBFNN. In order to avoid this problem, a normalization method is applied to convert all elements to the same magnitude.

The normalization method is expressed as

$$\begin{array}{c} x_{i}^{mag}=\max{\left\{x{\left(t_{0}\right)}_{i}\right\}}_{j=1}^{n}-\min{\left\{x{\left(t_{0}\right)}_{i}\right\}}_{j=1}^{n},i=1,2,\cdots 7 \end{array}$$ (21)

$$\begin{array}{c} \lambda_{i}^{mag}=\max{\left\{\lambda {\left(t_{0}\right)}_{i}\right\}}_{j=1}^{n}-\min{\left\{\lambda {\left(t_{0}\right)}_{i}\right\}}_{j=1}^{n},i=1,2\cdots 7 \end{array}$$ (22)

$$\begin{array}{c} \tilde{x}\left(t_{0}\right)=\{\tilde{x}{\left(t_{0}\right)}_{i}\}=\{\frac{x{\left(t_{0}\right)}_{i}-\min{\left\{x{\left(t_{0}\right)}_{i}\right\}}_{j=1}^{n}}{x_{i}^{mag}}\},i\in \arg\{x_{i}^{mag}\neq 0\} \end{array}$$ (23)

$$\begin{array}{c} \tilde{\lambda }\left(t_{0}\right)=\{\tilde{\lambda }{\left(t_{0}\right)}_{i}\}=\{\{\begin{array}{c} \frac{\lambda {\left(t_{0}\right)}_{i}-\min{\left\{\lambda {\left(t_{0}\right)}_{i}\right\}}_{j=1}^{n}}{\lambda_{i}^{mag}},\lambda_{i}^{mag}\neq 0\\ \;\text{0}\;,\lambda_{i}^{mag}=0 \end{array}\},i=1,2\cdots 7 \end{array}$$ (24)

where

$\tilde{\text{x}}(t_{0}),\tilde{\lambda }(t_{0})$ are the normalization vectors of the states and the co-states, respectively,

$x_{i}^{mag},\lambda_{i}^{mag}$ are the magnitude parameters

*n* is the number of the training data.

Through the normalization method, the magnitude of the initial states and co-states are located in [0, 1].

The inverse of the normalization method is expressed as

$$\begin{array}{c} \lambda \left(t_{0}\right)=\{\min{\left\{\lambda {\left(t_{0}\right)}_{i}\right\}}_{j=1}^{n}+\tilde{\lambda }{\left(t_{0}\right)}_{i}\lambda_{i}^{mag}\},i=1,2\cdots 7 \end{array}$$ (25)

#### The RBFNN

**Fig 3:**
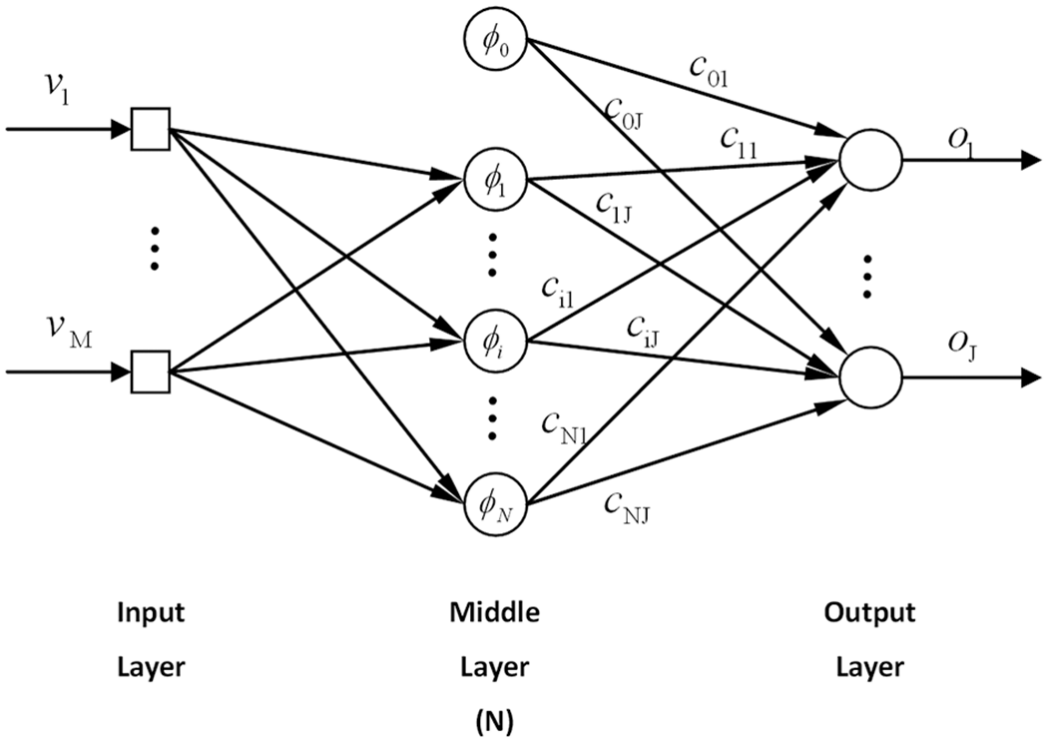
The Radial Basis Function Neural Network model. This figure illustrates the model of the RBFNN. The network has three layers: the input layer, the middle layer and the output layer. The input of the network is passed to all neurons by the input layer. In the middle layer, the neurons of the radial basis function output values based on the inputs. The output of the network is the sum of the outputs of the neurons with different coefficients by the output layer.

As illustrated in Fig 3, the output of the RBFNN is the sum of all outputs of the neurons with different coefficients. The center point of neurons is not necessarily structured, that is, it can have an arbitrary distribution. Such a mesh-free grid structure yields high flexibility, especially when the domain is irregular [27].

The output of RBF neurons are expressed as

$$\begin{array}{ccc} \varphi_{0}\left(v\right) & = & 1\\ \varphi_{j}\left(v\right) & = & e^{-\frac{{∥p_{j}-v∥}^{2}}{b^{2}}},j=1,\cdots N \end{array}$$ (26)

where

**v**, are the input of the RBF neural,

**p**_*j*_ is the center point of the RBF neuron,

*b* is the shape parameter,

The output of RBFNN can be expressed as

$$\begin{array}{c} o_{i}\left(v\right)=c_{0i}+\sum_{j=1}^{N-1}c_{ji}\varphi_{j}\left(v\right) \end{array}$$ (27)

where

*c* is the unknown coefficients,

*N* is the number of the neurons.

The training of the RBFNN is based on the orthogonal least squares. Define the error function as

$$\begin{array}{c} e_{ik}={\tilde{\lambda }}_{ik}-c_{0i}-\sum_{j=1}^{N-1}c_{ji}\varphi_{j}\left({\tilde{x}}_{k}\right),i=1,2,\cdots 7,k=1,2,\cdots n \end{array}$$ (28)

where

**e** is the 7 × *n* matrix of the error.

*n* is the number of the training data.

*N* is the number of the neurons.

Define the regression matrix as

$$\begin{array}{c} P\left(i\right)=[p{\left(i\right)}_{1},p{\left(i\right)}_{2},\cdots ,p{\left(i\right)}_{N}] \end{array}$$ (29)

$$\begin{array}{c} p{\left(i\right)}_{j}={\left[p{\left(i\right)}_{j1},p{\left(i\right)}_{j2},\cdots ,p{\left(i\right)}_{jn}\right]}^{T} \end{array}$$ (30)

$$\begin{array}{c} p_{jk}\left(i\right)=\varphi_{j}\left({\tilde{x}}_{k}\right) \end{array}$$ (31)

The orthogonal triangular decomposition of **P**(*i*) is expressed as

$$\begin{array}{c} P\left(i\right)=P_{ui}P_{ai} \end{array}$$ (32)

where

**P**_*ai*_ is a *N* × *N* Upper triangular matrix whose main diagonal elements are 1,

**P**_*ui*_ is a *n* × *N* Orthogonal matrix.

The weight vector **c**_*i*_ can be determined as below [28].

$$\begin{array}{c} c_{i}=P_{ai}^{-1}{\left(P_{ui}^{T}P_{ui}\right)}^{-1}P_{ui}{\left\{{\tilde{\lambda }}_{ik}\right\}}_{k=1}^{n} \end{array}$$ (33)

## IV. Simulation

Eros433, whose physical parameters are shown in Table 1, is selected to be the mission object in the simulation [29]. In the table, *G* is the gravitational constant, *M* is the total mass of the body, *R*_0_ is the normalizing radius of the asteroid, *C*_20_ and *C*_22_ are the spherical harmonic gravity coefficient, *ω* is the angular velocity of the asteroid’s rotation, *T*_max_ is the upper bound of the control thrust, *I*_*s*_ is the specific impulse of the thruster, *t*_*f*_ is the terminal time of the soft landing, *m*_0_ is the initial mass of the spacecraft. The second order of SHEM is applied as the gravity field model of the asteroid, its triaxial expressions are shown as Eqs (34)–(42).

**Table 1:** The parameters of the simulation.

parameter	value
*R*_0_/m	1.600e2
*GM*/(*km* ^3^/*s* ^2^)	3.068e-9
*C*_20_	5.247e-2
*C*_22_	8.253e-2
*ω*/(rad/s)	3.314e-4
*T*_max_/N	5.000e1
*I*_*s*_/s	4.000e2
*t*_*f*_/s	8.000e3
*m*_0_/kg	2.000e3

The gravitational acceleration in three axis are expressed as

$$\begin{array}{c} g_{x}=-\frac{GMx}{r^{3}}[1+\frac{3}{2}C_{20}{\left(\frac{R_{0}}{r}\right)}^{2}(5\frac{z^{2}}{r^{2}}-1)+3C_{22}{\left(\frac{R_{0}}{r}\right)}^{2}(5\frac{x^{2}-y^{2}}{r^{2}}-2)] \end{array}$$ (34)

$$\begin{array}{c} g_{y}=-\frac{GMy}{r^{3}}[1+\frac{3}{2}C_{20}{\left(\frac{R_{0}}{r}\right)}^{2}(5\frac{z^{2}}{r^{2}}-1)+3C_{22}{\left(\frac{R_{0}}{r}\right)}^{2}(5\frac{x^{2}-y^{2}}{r^{2}}+2)] \end{array}$$ (35)

$$\begin{array}{c} g_{z}=-\frac{GMz}{r^{3}}[1+\frac{3}{2}C_{20}{\left(\frac{R_{0}}{r}\right)}^{2}(5\frac{z^{2}}{r^{2}}-3)+15C_{22}{\left(\frac{R_{0}}{r}\right)}^{2}(\frac{x^{2}-y^{2}}{r^{2}})] \end{array}$$ (36)

The gravitational gradient are expressed as

$$\begin{array}{c} \frac{\partial^{2}U}{\partial x^{2}}=\frac{\partial U}{\partial xx}+3\frac{GMx^{2}}{r^{5}}[1+\frac{5}{2}C_{20}{\left(\frac{R_{0}}{r}\right)}^{2}(7\frac{z^{2}}{r^{2}}-1)+5C_{22}{\left(\frac{R_{0}}{r}\right)}^{2}(7\frac{x^{2}-y^{2}}{r^{2}}-4)] \end{array}$$ (37)

$$\begin{array}{c} \frac{\partial^{2}U}{\partial y^{2}}=\frac{\partial U}{\partial yy}+3\frac{GMy^{2}}{r^{5}}[1+\frac{5}{2}C_{20}{\left(\frac{R_{0}}{r}\right)}^{2}(7\frac{z^{2}}{r^{2}}-1)+5C_{22}{\left(\frac{R_{0}}{r}\right)}^{2}(7\frac{x^{2}-y^{2}}{r^{2}}+4)] \end{array}$$ (38)

$$\begin{array}{c} \frac{\partial^{2}U}{\partial z^{2}}=\frac{\partial U}{\partial zz}+3\frac{GMz^{2}}{r^{5}}[1+\frac{5}{2}C_{20}{\left(\frac{R_{0}}{r}\right)}^{2}(7\frac{z^{2}}{r^{2}}-5)+35C_{22}{\left(\frac{R_{0}}{r}\right)}^{2}(\frac{x^{2}-y^{2}}{r^{2}})] \end{array}$$ (39)

$$\begin{array}{c} \frac{\partial^{2}U}{\partial x\partial y}=\frac{\partial^{2}U}{\partial y\partial x}=3\frac{GMxy}{r^{5}}[1+\frac{5}{2}C_{20}{\left(\frac{R_{0}}{r}\right)}^{2}(7\frac{z^{2}}{r^{2}}-1)+35C_{22}{\left(\frac{R_{0}}{r}\right)}^{2}(\frac{x^{2}-y^{2}}{r^{2}})] \end{array}$$ (40)

$$\begin{array}{c} \frac{\partial^{2}U}{\partial x\partial z}=\frac{\partial^{2}U}{\partial z\partial x}=3\frac{GMxz}{r^{5}}[1+\frac{5}{2}C_{20}{\left(\frac{R_{0}}{r}\right)}^{2}(7\frac{z^{2}}{r^{2}}-3)+5C_{22}{\left(\frac{R_{0}}{r}\right)}^{2}(7\frac{x^{2}-y^{2}}{r^{2}}-2)] \end{array}$$ (41)

$$\begin{array}{c} \frac{\partial^{2}U}{\partial y\partial z}=\frac{\partial^{2}U}{\partial z\partial y}=3\frac{GMyz}{r^{5}}[1+\frac{5}{2}C_{20}{\left(\frac{R_{0}}{r}\right)}^{2}(7\frac{z^{2}}{r^{2}}-3)+5C_{22}{\left(\frac{R_{0}}{r}\right)}^{2}(7\frac{x^{2}-y^{2}}{r^{2}}+2)] \end{array}$$ (42)

It is assumed that the descending of the spacecraft starts at a random hovering point in a certain space and ends at a desired hovering point near the surface.

### A. The descending with fixed optimal control

**Fig 4:**
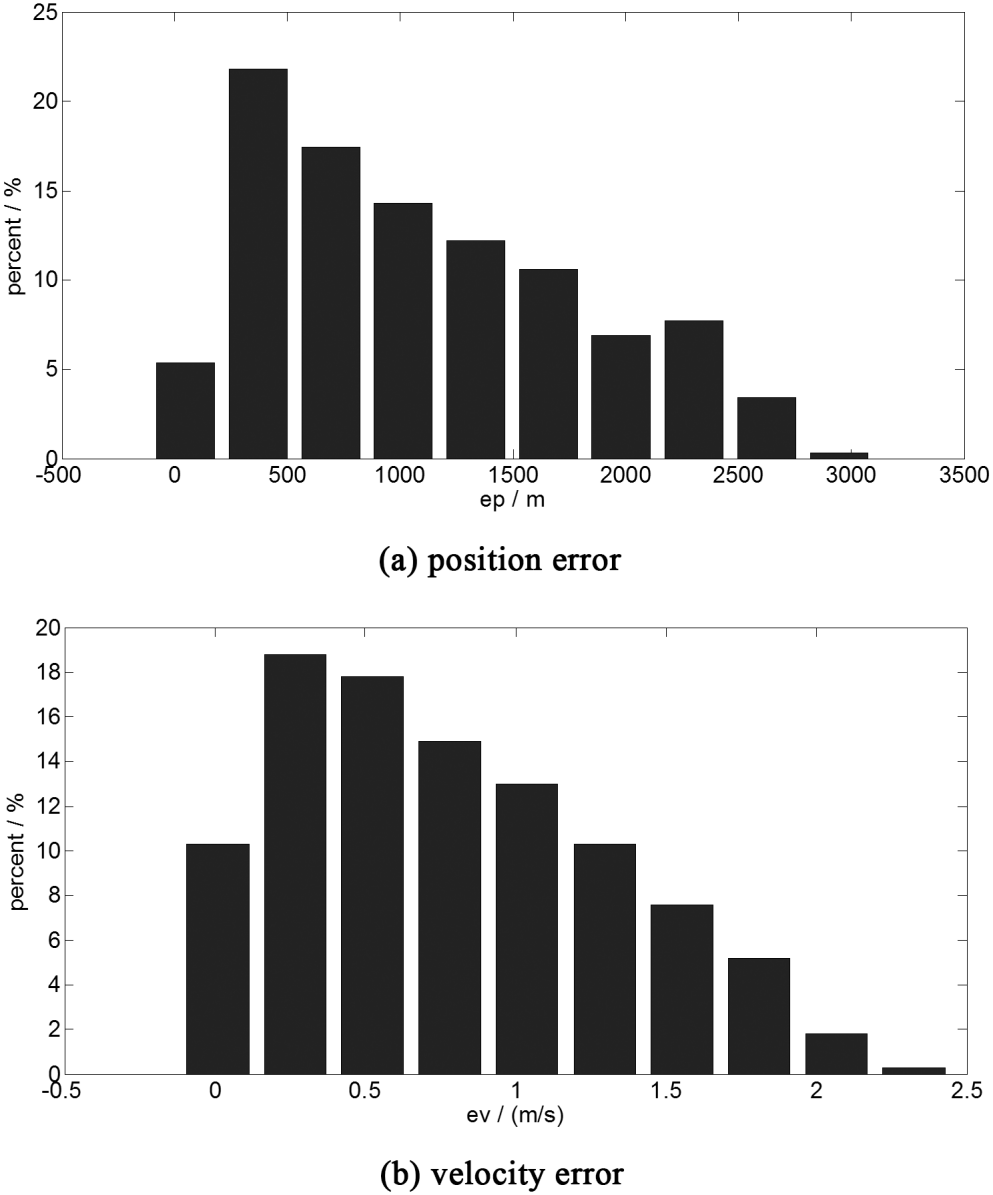
The terminal error histogram (fixed optimal control). a:position error. b:velocity error.This figure shows the results of the one thousand time simulations with the fixed optimal control. The abscissa is the error range, the ordinate is the statistical probability of the different ranges of the error.

A Monte Carlo simulation (one thousand times) is applied as the normal group to clarify the necessity of the online optimal guidance. In the simulation A, the spacecraft hovers in a random point in the space of [0 ± 0.1, 70 ± 0.1, 0 ± 0.1] km at the start. The fixed optimal control of the initial point [0, 70, 0] km is used for the soft landing. The statistical results of the simulation are shown in Fig 4.

Define the terminal error of position and velocity as

$$\begin{array}{c} e_{p}={∥x\left(t_{f}\right)-17000,y\left(t_{f}\right),z\left(t_{f}\right)∥}_{2} \end{array}$$ (43)

$$\begin{array}{c} e_{v}={∥\dot{x}\left(t_{f}\right),\dot{y}\left(t_{f}\right),\dot{z}\left(t_{f}\right)∥}_{2} \end{array}$$ (44)

As illustrated in Fig 4a, the magnitude of the terminal position error under the fixed optimal control is km level. The max terminal position error is about 3 km. The magnitude of the terminal velocity error is meter per second level, which is shown in Fig 4b. The average error of position and velocity are 1099.1 meters and 0.7873 m/s, respectively. These results are not acceptable for the autonomous guidance. Hence, the precomputed optimal control cannot guarantee the safety of the soft landing. The autonomous optimal guidance is necessary.

### B: The descending with RBFNN (150 neurons trained by 1331 sets of data)

Firstly, the optimal soft landing trajectories about 1331 initial hovering points, which have an interval of 1000m between the adjacent two points, are selected to establish the training database.Then, a RBFNN of 150 neurons is trained by the database. Finally, the network is applied in the simulation of the online optimal guidance.

A Monte Carlo simulation (one thousand times) is conducted to verify the proposed optimal guidance. The spacecraft is assumed to be hovering on a random point in the selected space of [0 ± 5, 70 ± 5, 0 ± 5] km at the start, and then descend to the desired hovering point applying the proposed optimal guidance..

**Fig 5:**
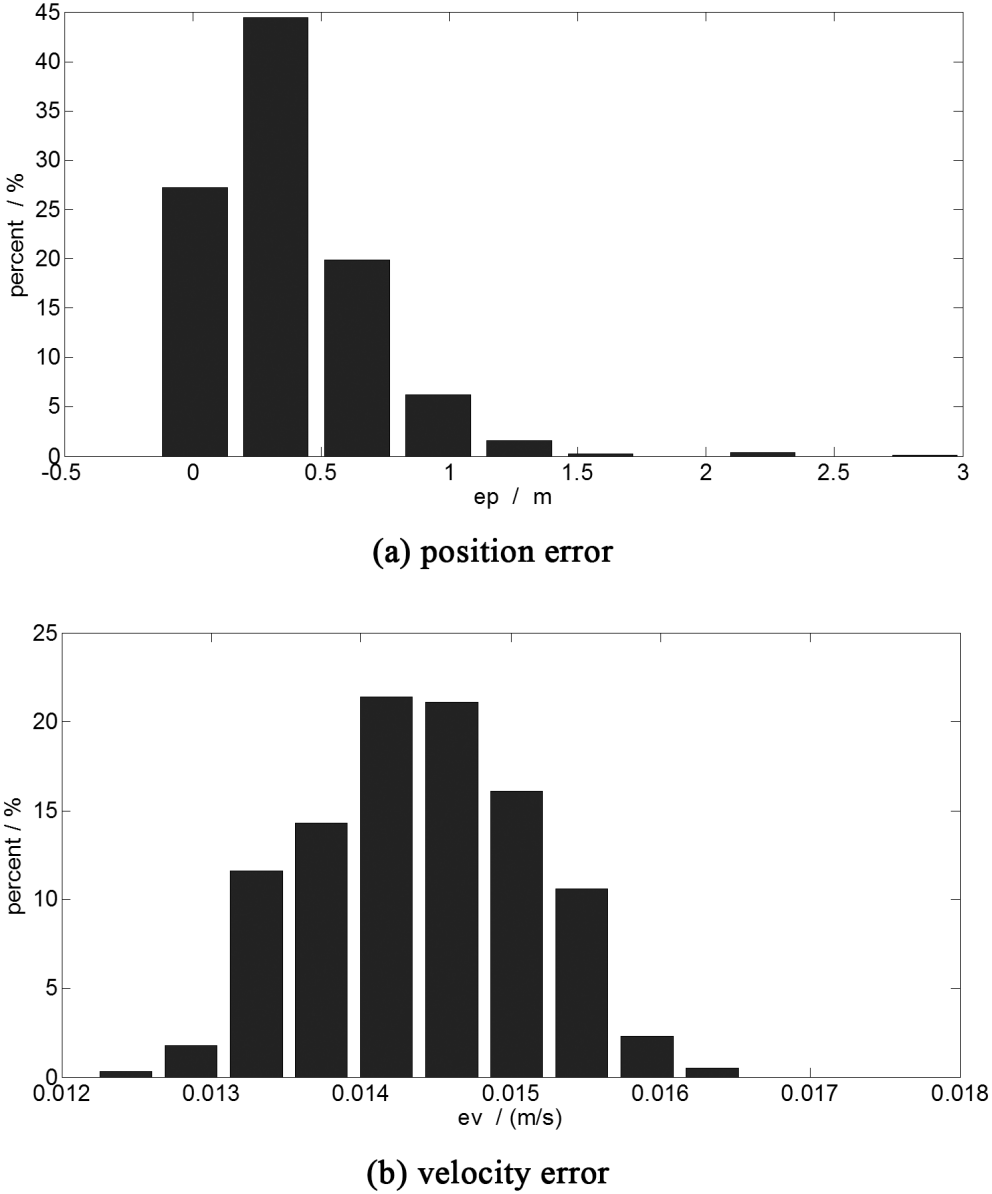
The terminal error histogram (150 neurons trained by 1331 sets of data). a:position error. b:velocity error.This figure shows the results of the one thousand time simulations with the fast optimal control of 150 neurons trained by the database of 1331 sets of data. The abscissa is the error range, the ordinate is the statistical probability of the different ranges of the error.

The statistical results of the simulation are shown in Fig 5.

As illustrated in Fig 5a, more than ninety percent of terminal position errors in the simulation is less than 1 meter. The max terminal position error is less than 3 meters. The most of the terminal velocity errors are less than 0.16 m/s, which are shown in Fig 5b. The initial random space of the spacecraft is 1000 *km* ^3^, which is 125000 times larger than the space in the simulation A. The average position error is 0.3779 meters, which is 1/2900 of the results in Fig 4. The average velocity error is 0.0144 m/s, which is 1/54 of the results in Fig 4. The simulation shows that the proposed optimal guidance is better than the fixed optimal guidance.

The contrast of the two obtained co-states $\lambda_{i}^{s}$ and $\lambda_{i}^{\text{rbf}}$ are shown in Table 2. The initial co-state $\lambda_{i}^{s}$ is acquired through solving the shooting equations. The initial co-state $\lambda_{i}^{\text{rbf}}$ is acquired through the trained RBFNN. The initial state **x**_1_ is a sample point in the training database. The **x**_2_ is a random point in the selected space, but not in the samples.

**Table 2:** The contrast of the results.

x_1_	$\lambda_{1}^{s}$	$\lambda_{1}^{\text{rbf}}$	x_2_	$\lambda_{2}^{s}$	$\lambda_{2}^{\text{rbf}}$
-5000	1.143020e0	1.143021e0	629.45	1.019031e0	1.019031e0
75000	4.087609e-1	4.087611e-1	70812	3.822612e-1	3.822610e-1
3000	1.243641e-2	1.243657e-2	-746.03	3.114120e-3	3.114120e-3
0	-1.570168e3	-1.570168e3	0	-1.481219e3	-1.481218e3
0	1.366568e3	1.366568e3	0	1.321191e3	1.321190e3
0	4.497661e1	4.497717e1	0	-1.118024e1	-1.118024e1
2000	8.054259e1	8.054265e1	2000	6.867938e1	6.867930e1

Contrasting the results shown in Table 2, each element in the $\lambda_{i}^{\text{rbf}}$ is similar to the ones in the $\lambda_{i}^{s}$, no matter at the selected point or random point. However, the computation time of the RBFNN is 12.04 milliseconds in the environment of MATLAB with a 2.59 GHz CPU. It is much faster than the traditional indirect method based on Newton’s or Powell’s, which may need minutes even hours [8].

### C: The descending with RBFNN (1000 neurons trained by 1331 sets of data)

**Fig 6:**
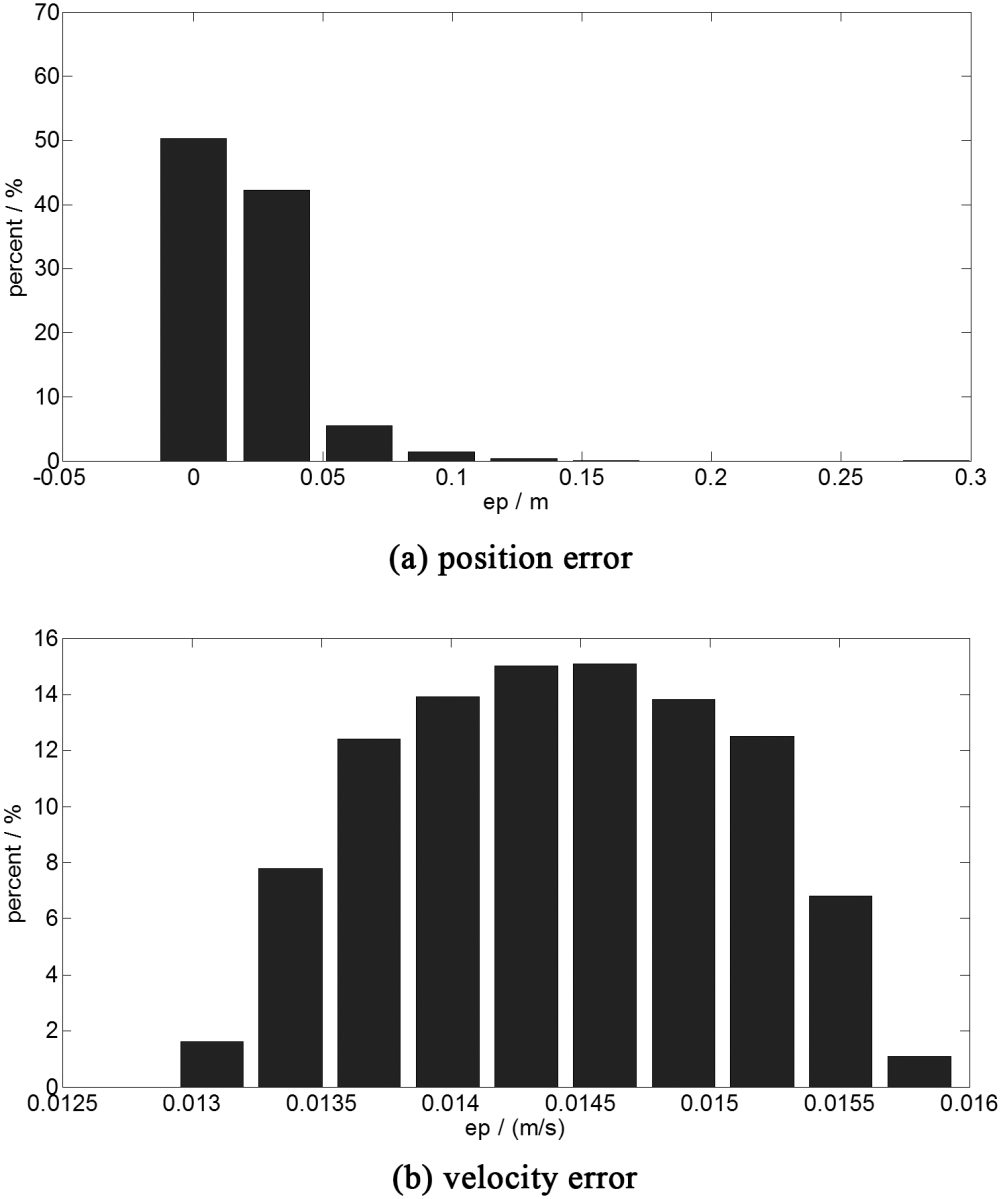
The terminal error histogram (1000 neurons trained by 1331 sets of data)). a:position error. b:velocity error. This figure shows the results of the one thousand time simulations with the fast optimal control of 1000 neurons trained by the database of 1331 sets of data. The abscissa is the error range, the ordinate is the statistical probability of the different ranges of the error.

Aiming to improve the accuracy of the proposed optimal guidance, the RBFNN is extended to 1000 neurons. The results of the Monte Carlo simulation are shown in Fig 6

As illustrated in Fig 6a, the max terminal position error is reduced to 0.3 meters and the average value is reduced to 0.0214 meters. The velocity error is not reduced much, but it is more concentrated. Compare with the results in Fig 5 and Fig 6, it is easy to find that the accuracy of the optimal guidance law is improved with the increasing of the neurons.

### D: The descending with RBFNN (1000 neurons trained by 9261 sets of data)

**Fig 7:**
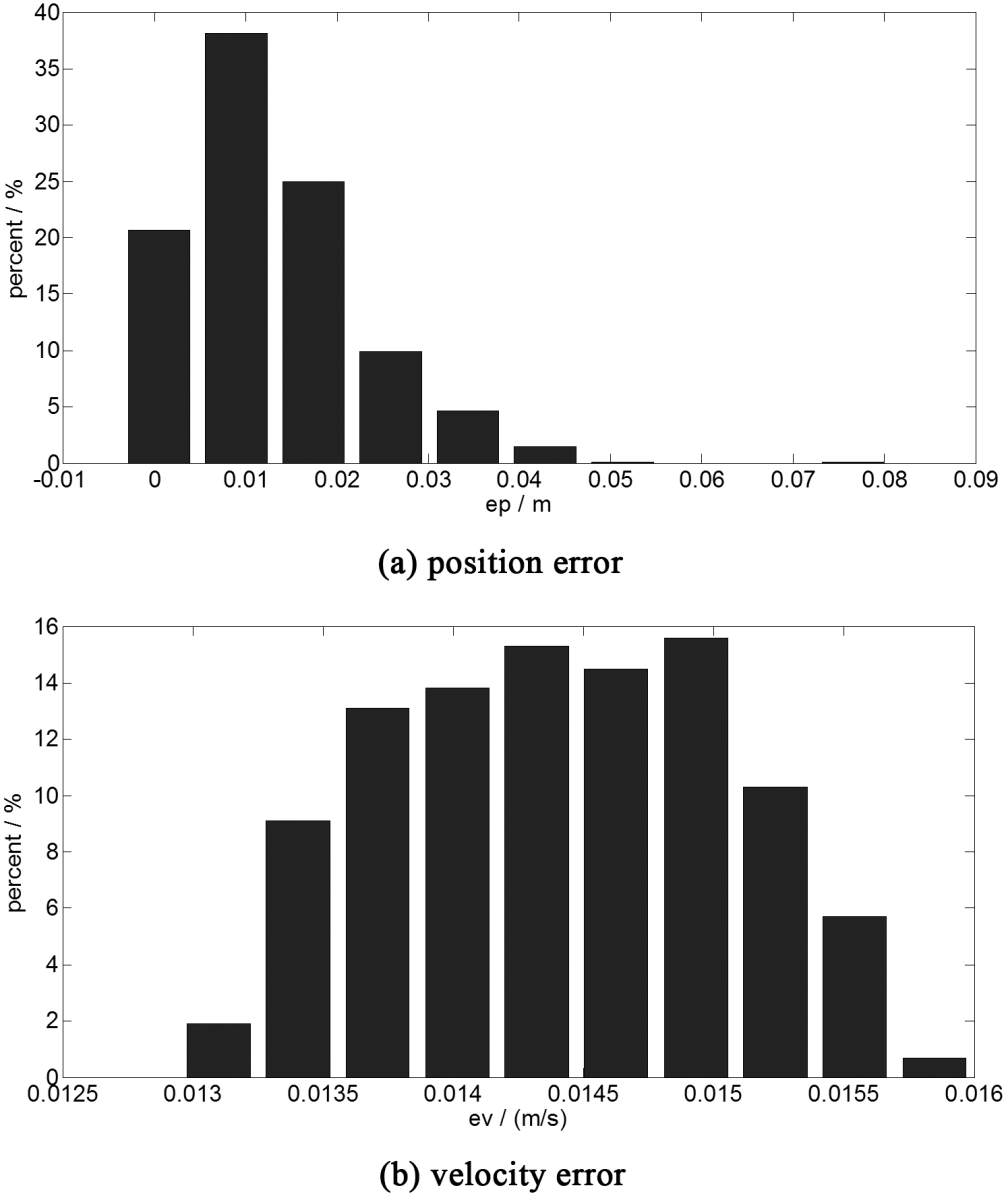
The terminal error histogram (1000 neurons trained by 9261 sets of data)). a:position error. b:velocity error. This figure shows the results of the one thousand time simulations with the fast optimal control of 1000 neurons trained by the database of 9261 sets of data. The abscissa is the error range, the ordinate is the statistical probability of the different ranges of the error.

To improve the performance of the proposed guidance further, the trained database is extended to 9261 sets of data. The results of Monte Carlo are shown in Fig 7.

The maximum of the terminal position error is reduced to 0.077 meters and the average value is reduced to 0.013 meters. which is shown in Fig 7a. With the extended database, the accuracy of the proposed guidance algorithm is improved further.

As a conclusion of the simulations above, it is obvious that the proposed optimal guidance is effective. It can guide the spacecraft to the desired point. The computing time is only a few milliseconds. The accuracy of the proposed optimal guidance can be improved by increasing the number of the neurons and the training data set.

## V. Conclusion

This paper proposed a fast and computationally inexpensive optimal guidance algorithm via RBFNN for the autonomous soft landing on an asteroid. The well trained RBFNN was applied online to determine the optimal soft landing trajectory without solving the shooting equation, which leads the computing time of the optimal guidance law reduced to a few milliseconds. The accuracy of the proposed guidance algorithm can be improved through the extending of the network and the training database. Hence, it can meet the requirements of the practical if the network and database are appropriate. As a consequence, the proposed optimal guidance algorithm can guide the spacecraft to the desired point. Also, it is fast and computationally inexpensive enough to be selected in the autonomous soft landing task.

Several limitations still exist in our work. The proposed method focuses on reducing the online cost of optimal guidance, the offline cost of the pre-computation process is still very high. The outside disturbance on the spacecraft during the soft landing needs a further research. The training process of the network can also be improved. These works will be done in the future.

## Supporting Information

S1 Mat Training data(1331 sets).mat (MAT)

S2 Mat Training data(9261 sets).mat (MAT)

S3 Mat The RBFNN of 150 nodes trained by 1331 sets of data.mat (MAT)

S4 Mat The RBFNN of 1000 nodes trained by 1331 sets of data.mat (MAT)

S5 Mat The RBFNN of 1000 nodes trained by 9261 sets of data.mat (MAT)

S6 Mat Results of Monte Carlo simulations-150-1331.mat (MAT)

S7 Mat Results of Monte Carlo simulations-1000-1331.mat (MAT)

S8 Mat Results of Monte Carlo simulations-1000-9261.mat (MAT)
